# Antihistamines H_1_ as Potential Anthelmintic Agents against the Zoonotic Parasite *Angiostrongylus cantonensis*

**DOI:** 10.1021/acsomega.4c04773

**Published:** 2024-07-02

**Authors:** Daniel B. Roquini, Bruna L. Lemes, Amanda L. B. Kreutz, Sophia C. Spoladore, Monique C. Amaro, Flavia B. Lopes, João Paulo S. Fernandes, Josué de Moraes

**Affiliations:** † Núcleo de Pesquisa em Doenças Negligenciadas, Universidade Guarulhos, 07023-070 Guarulhos, SP, Brazil; ‡ Departamento de Medicina, Universidade Federal de São Paulo, 04023-062 São Paulo, SP, Brazil; § Departamento de Ciências Farmacêuticas, Universidade Federal de São Paulo, 09913-030 Diadema, SP, Brazil; ∥ Núcleo de Pesquisa em Doenças Negligenciadas, Instituto Científico e Tecnológico, Universidade Brasil, 08230-030 São Paulo, SP, Brazil

## Abstract

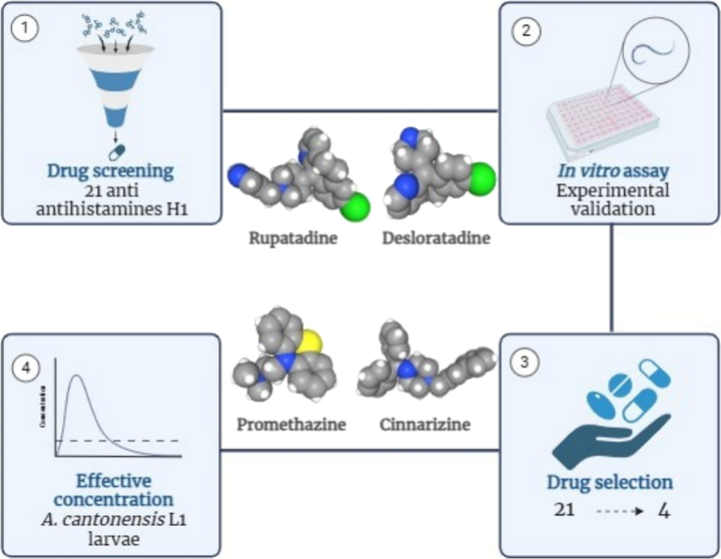

Infections caused by parasitic helminths pose significant health concerns for both humans and animals. The limited efficacy of existing drugs underscores the urgent need for novel anthelmintic agents. Given the reported potential of antihistamines against various parasites, including worms, this study conducted a screening of clinically available antihistamines against *Angiostrongylus cantonensis*—a nematode with widespread implications for vertebrate hosts, including humans. Twenty-one *anti*-H_1_ antihistamines were screened against first-stage larvae (L_1_) of *A. cantonensis* obtained from the feces of infected rats. Standard anthelmintic drugs ivermectin and albendazole were employed for comparative analysis. The findings revealed four active compounds (promethazine, cinnarizine, desloratadine, and rupatadine), with promethazine demonstrating the highest potency (EC_50_ = 31.6 μM). Additionally, morphological analysis showed that antihistamines induced significant changes in larvae. To understand the mechanism of action, antimuscarinic activities were reported based on average p*K*_i_ values for human muscarinic receptor (mAChR) subtypes of the evaluated compounds. Furthermore, an analysis of the physicochemical and pharmacodynamic properties of antihistamines revealed that their anthelmintic activity does not correlate with their activity at H1 receptors. This study marks the first documentation of antihistamines’ activity against *A. cantonensis*, offering a valuable contribution to the quest for novel agents effective against zoonotic helminths.

## 1 Introduction

Parasitic worm infections represent a significant yet often underestimated burden on both human and animal health worldwide. Particularly prevalent in tropical and subtropical regions, these infections disproportionately affect impoverished and marginalized communities with limited access to clean water and adequate sanitation.^1^ Global estimates indicate that around 1.5 billion individuals are afflicted with at least one parasitic worm,^2^ with many emerging infectious diseases being of zoonotic origin.^3^ Recognizing the gravity of these conditions, the World Health Organization (WHO) launched a new roadmap in 2021 aimed at combating neglected diseases, including helminthiasis, by 2030.^4^ Despite these efforts, the arsenal of recommended anthelmintics remains limited.^5,6^

Among the diverse array of parasitic worms, *Angiostrongylus cantonensis*, commonly known as the rat lungworm, stands out as a nematode belonging to the Metastrongyloidea superfamily. Its larval stages develop primarily in various mollusks, particularly snails and slugs, before potentially infecting a range of vertebrate hosts.^7^ In humans, *A. cantonensis* infection often manifests as eosinophilic meningitis, with larvae infiltrating the brain and eliciting local host responses. Symptoms typically include headaches, fever, malaise, and various neurological impairments, occasionally leading to fatal outcomes.^8^ Ocular angiostrongyliasis, characterized by uveitis and vision loss, further underscores the severity of human infections. Reported cases of human angiostrongyliasis span across continents, including Africa, Southeast Asia, Oceania, South America, the Caribbean, the United States, and Europe.^9^ The documented occurrence of angiostrongyliasis among travelers underscores the necessity of strategies to mitigate the emergence of zoonotic pathogens.^10,11^

Despite *A. cantonensis* being the primary cause of eosinophilic meningitis globally, epidemiological data are limited. Approximately 3000 cases have been reported globally, but additional records suggest that the number is at least 7000.^12^ Many more cases likely go unreported because symptoms are mild and short-lived, leading people not to seek medical help, or because the disease is misdiagnosed. A pilot seroepidemiological study in Hawaii in 2015 supports this finding that 22% of 435 donated blood samples tested positive for *A. cantonensis* antibodies.^13^ Treatment is typically supportive, using painkillers and corticosteroids to reduce inflammation. No anthelmintic drugs have been proven effective in the treatment.^12^

The inadequacies of current anthelmintics highlight the pressing need for novel therapeutic agents. Given its ability to infect both animals and humans, coupled with its easily maintainable life cycle in laboratory rodents, *A. cantonensis* serves as a compelling model for anthelmintic research.^14^ Despite its neglected status in terms of research investment, drug repurposing emerges as a promising avenue for discovering novel therapeutic interventions. Drug repurposing involves exploring existing drugs for new therapeutic applications, offering a rapid, cost-effective, and low-risk strategy, particularly for neglected diseases.^15,16^ Notably, repurposed drugs benefit from extensive safety data, streamlining the clinical approval process and leveraging existing pharmaceutical supply chains.^17,18^

Antihistamines have garnered attention as promising candidates in drug repurposing endeavors, including their potential as antiparasitic agents.^16^ Recent research from our group demonstrated the efficacy of antihistamines against *Schistosoma mansoni* worms,^19,20^ prompting further exploration of their potential against *A. cantonensis*. In this study, we investigated the anthelmintic potential of a selection of clinically available antihistamines against the first larval (L1) stages of *A. cantonensis*. These findings suggest that antihistamines may represent not only novel agents against this parasite but also potential prototypes for further refinement in the pursuit of new anthelmintics within drug discovery programs.

## 2 Results and Discussion

**Figure 1:**
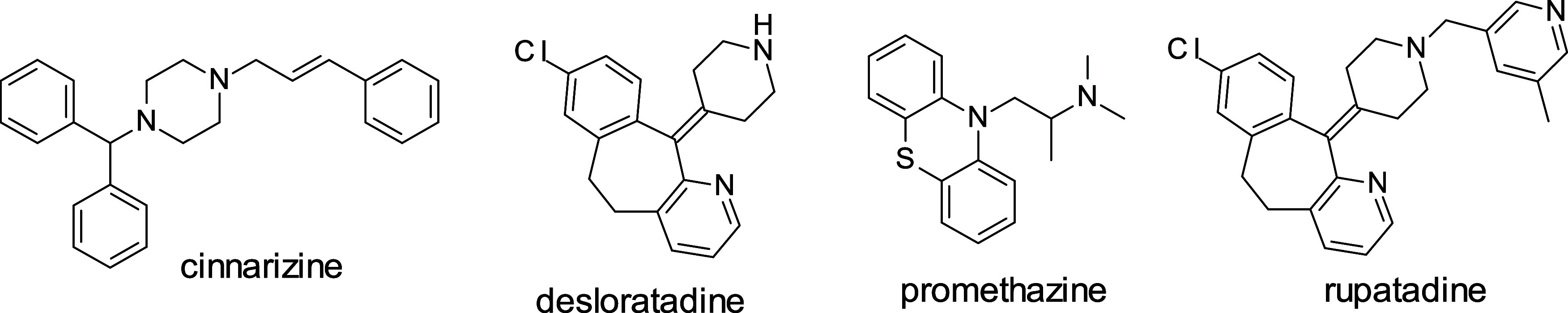
Structural representation of the antihistamines with activity against *A. cantonensis* L1 larvae.

**Figure 2:**
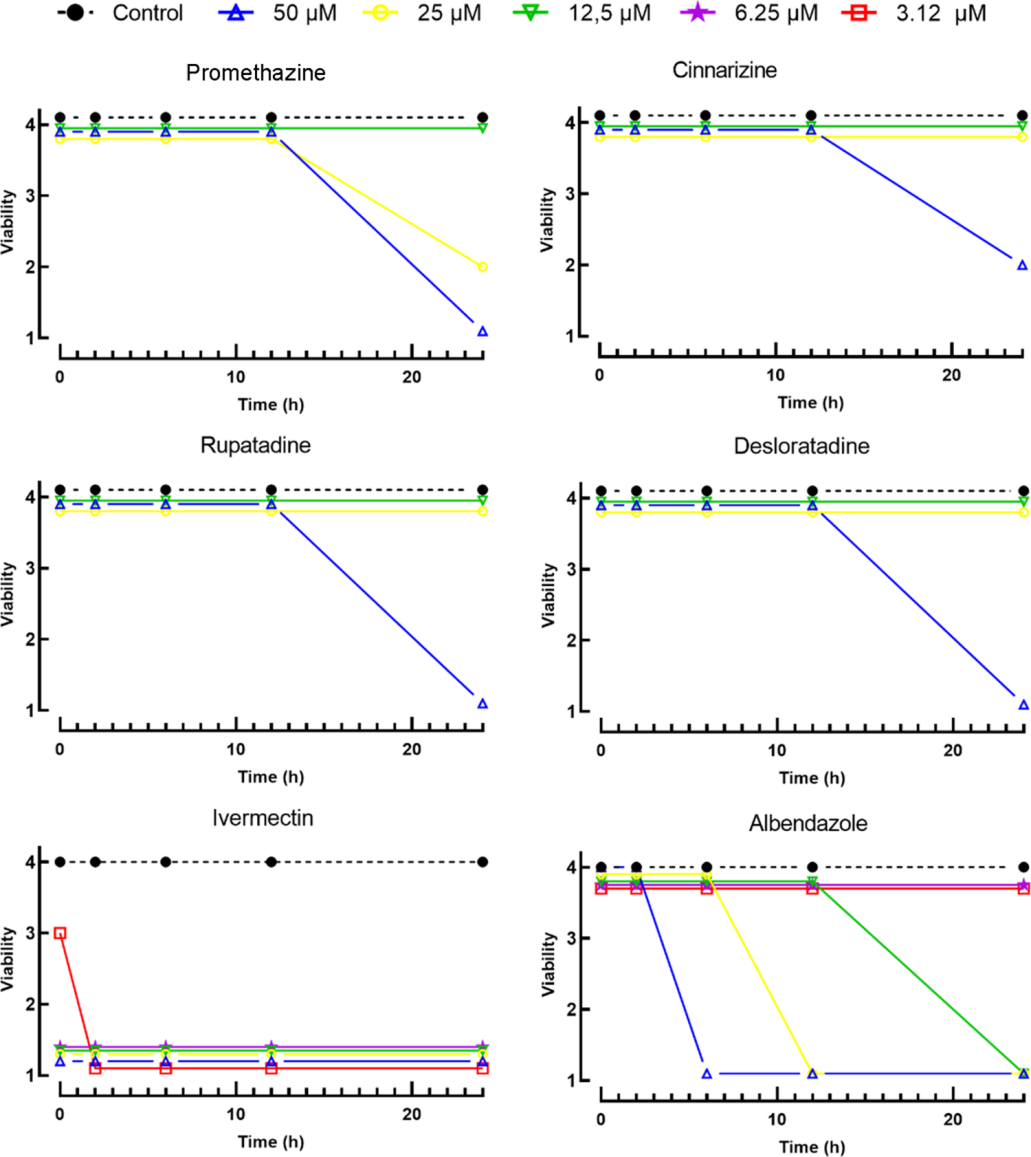
Time-dependent effects of promethazine, cinnarizine, rupatadine, and desloratadine at various concentrations against *A. cantonensis* L1 larvae. Larval viability was assessed at indicated time points using the following scoring system: 1 (immotile), 2 (intermittent shaking of the head or tail region), 3 (sluggish and motile), or 4 (highly active and motile).

The larval motility assay stands as the preferred method for evaluating drug sensitivity across various nematode species,^21−23^ including *A. cantonensis*.^14^ Among the antihistamines tested, four (cinnarizine, desloratadine, promethazine, and rupatadine, Figure 1) were found to impact larval viability at 50 μM. These compounds underwent further testing to ascertain their EC_50_ values against *A. cantonensis* L1 larvae. As depicted in Table 1 and Figure 2, the drugs exhibited concentration- and time-dependent effects.

**Table 1:** Anthelmintic Activity of the Selected Antihistamines against *A. cantonensis* L1 Larvae

drug	EC_50_ (μM, ± SD)	drug	EC_50_ (μM, ± SD)
acrivastine	>100	hydroxyzine	>100
astemizole	>100	ketotifen	>100
bilastine	>100	levocetirizine	>100
carebastine	>100	loratadine	>100
cetirizine	>100	meclizine	>100
chlorfeniramine	>100	mizolastine	>100
cinnarizine	45.9 ± 6.3	promethazine	31.6 ± 5.1
desloratadine	34.2 ± 5.6	rupatadine	38.1 ± 6.4
dexchlorpheniramine	>100	terfenadine	>100
epinastine	>100	tripelennamine	>100
fexofenadine	>100		
ivermectin	1.6 ± 0.5	albendazole	11.3 ± 1.4

The active antihistamines demonstrated EC_50_ values ranging from 31.6 to 45.9 μM, with promethazine exhibiting the highest potency (Table 1). In comparison, standard anthelmintics ivermectin and albendazole demonstrated anthelmintic effects with EC_50_ values of 1.6 and 11.3 μM, respectively. Temporal analysis of the activity of promethazine, cinnarizine, rupatadine, and desloratadine against L1 larvae revealed that the anthelmintic effect of these antihistamines on *A. cantonensis* manifested only after 24 h of exposure to 50 μM of each drug. In contrast, ivermectin, a widely utilized anthelmintic drug, induced larval paralysis within the first 2 h at a concentration of 3.12 μM, with complete immobilization observed at 6.25 μM. Similarly, albendazole exhibited a slightly delayed onset of action, with maximal loss of spontaneous movement observed at 12.5 μM within 2 and 24 h, respectively, consistent with previous studies.^14^ However, the antihistamines required a longer duration and higher concentrations to achieve comparable effects, indicating a distinct mode of action compared to clinically available anthelmintics.

**Figure 3:**
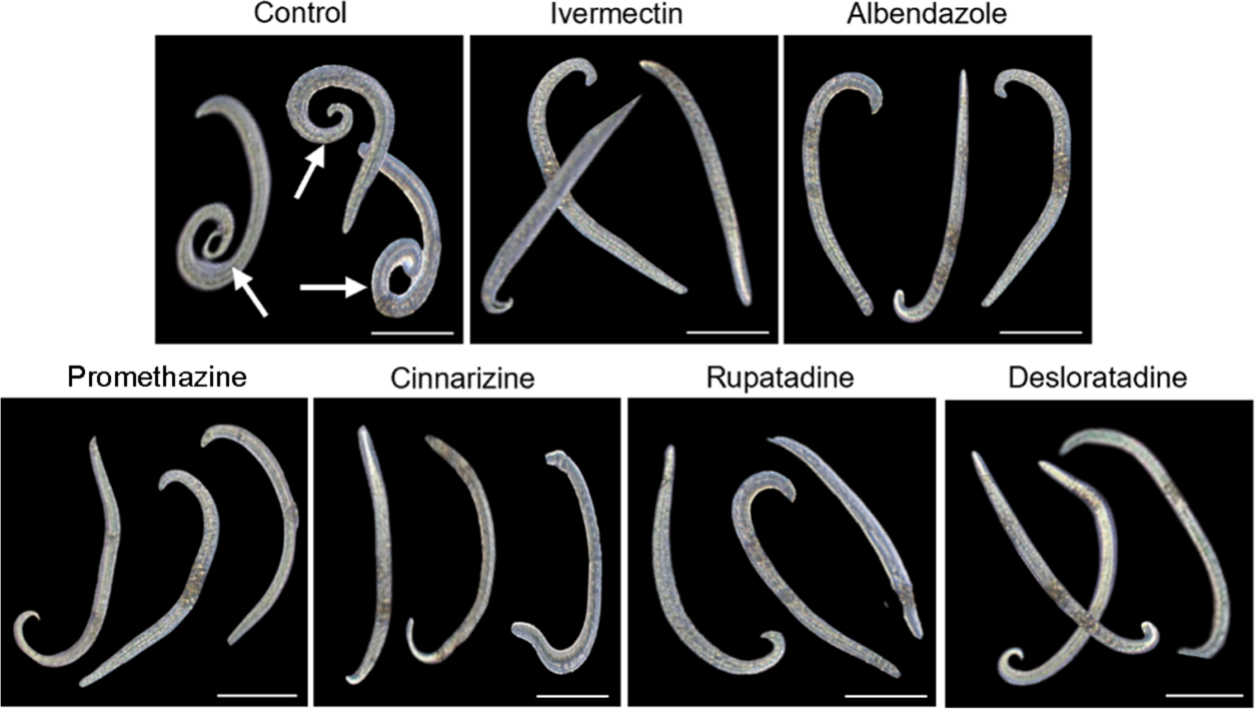
Morphological analysis of *A. cantonensis* L1 larvae during incubation with antihistamines and control worms. Larvae in the negative control group exhibited contortion in their caudal region (arrow). Antihistamines promethazine, cinnarizine, rupatadine, and desloratadine were tested at 50 μM, while ivermectin and albendazole, anthelmintic drugs used as positive controls, were tested at 3.15 and 12.5 μM, respectively. Images were captured using a Motic AE2000 inverted microscope equipped with a Motic ultrahigh definition camera (scale bars = 23 μm).

Furthermore, morphological analysis using light microscopy revealed that antihistamines induced morphological changes in larvae compared to the negative control group (Figure 3). Specifically, akin to the classical anthelmintic drugs ivermectin and albendazole, worms treated with antihistamines did not display contortion in their caudal region, indicating distinct phenotypic variations between worms exposed to antihistamines and controls.

Histamine, a pleiotropic biogenic amine, exerts diverse effects in mammals, including modulation of immune and inflammatory responses, as well as regulation of central nervous system functions.^24^ While invertebrates typically lack metabotropic histamine receptors, they may express an ionotropic histamine receptor, HisCl, akin to the GABA-A receptor, which, upon histamine activation, enhances chloride ion permeability.^25,26^ Notably, anthelmintics’ activity on *A. cantonensis* did not correlate with their activity at H_1_ receptors. Potent antihistamines such as dexchlorpheniramine and ketotifen failed to exhibit activity against *A. cantonensis*, suggesting that the ability to bind to histamine receptors may not be implicated. Antihistamines are often described as “promiscuous” drugs, capable of binding to diverse pharmacological targets beyond H_1_ receptors, particularly receptors for other biogenic amines. For instance, desloratadine and promethazine exhibit considerable potency at muscarinic receptors, while astemizole and ketotifen display lower potency at these receptors.^27,28^ This suggests that activity at muscarinic receptors may underlie their observed anthelmintic activity (Table 2).

**Table 2:** Antihistaminic and Antimuscarinic Activities of the Evaluated Compounds^a^

drug	p*K*_i_ H_1_R^b^	affinity mAChR	drug	p*K*_i_ H_1_R^b^	affinity mAChR
acrivastine	n.a.	n.a.	hydroxyzine	8.7	+^c^
astemizole	8.5	+^b^	ketotifen	9.8	++^b^
bilastine	7.2	n.a.	levocetirizine	8.5	n.a.
carebastine	n.a.	n.a.	loratadine	6.4	n.a.
cetirizine	7.1	n.a.	meclizine	6.9^c^	+^c^
chlorfeniramine	8.2	++^c^	mizolastine	8.6	-
cinnarizine	8.1	++^b^	promethazine	9.5	++++^b^
desloratadine	8.8	+++^b^	rupatadine	8.4	n.a.
dexchlorpheniramine	9.1	++^c^	terfenadine	7.2	+^b^
epinastine	7.6	n.a.	tripelennamine	8.0	+^c^
fexofenadine	7.6	n.a.			

a The antimuscarinic activity was defined based on average p*K*_i_ values for human muscarinic receptors (mAChR) subtypes and are coded as follows: p*K*_i_ < 5 (0); p*K*_i_ 5-6 (+); p*K*_i_ 6-7 (++); p*K*_i_ 7-8 (+++); and p*K*_i_ > 8 (++++).

b Retrieved from Guide to Pharmacology database.^28^

c Obtained from Kubo et al.,^27^ n.a.: not available.

Acetylcholine serves as a primary neurotransmitter in nematodes, regulating muscle contraction and other physiological functions. While cholinergic nicotinic receptors represent classical anthelmintic targets, the existence and role of muscarinic receptors in worms remain unclear. Nematodes purportedly express G-protein acetylcholine receptors (GAR-1 to GAR-3), with GAR-3 bearing resemblance to vertebrate muscarinic receptors.^29,30^ The activity of antihistamines on these receptors warrants further investigation, though the observed paralytic effect of active antihistamines and the excitatory role attributed to cholinergic effects suggest a potential relationship with anticholinergic activity. Subsequent studies are needed to validate this hypothesis.

The active antihistamines are divided into two distinct classes of compounds: sedatives (first-generation) and nonsedatives (second-generation).^31^ The first-generation antihistamines are known to cross the blood–brain barrier (BBB) and inhibit the histamine H_1_ receptor in the CNS, leading to sedating effects. On the other hand, newer antihistamines are considerably less sedating due to lower lipophilicity and/or efflux by P-glycoprotein (Pgp), which leads to lower penetration into the CNS.

The analysis of the predicted physicochemical and pharmacokinetic properties (Table 3) corroborates this since these antihistamine are lipophilic compounds (*c* log* P* values), even considering their ionization in the physiological pH (*c* log *D* values). However, both desloratadine and rupatadine do not cross the BBB for being subject to efflux by Pgp.^31−33^ In counterpart, cinnarizine and promethazine are sedatives, denoting their ability to cross the BBB.^34^ The TPSA is another descriptor correlated to BBB permeation since the higher the TPSA value, the more hydrophilic the molecule is.^35^ Literature reports also support that TPSA better describes the ability to cross the BBB.^36^ As can be noted, cinnarizine and promethazine present low TPSA values, while the nonsedating drugs desloratadine and rupatadine showed higher TPSA values.

**Table 3:** Selected Physicochemical Properties and Pharmacodynamics of the Active Antihistamines

drug	*c *log *P*	*c* log *D*_7.4_	TPSA	promiscuity^a^	additional targets^b^
cinnarizine	4.72	3.95	6.5	0.953	Ca^2+^ channels, cholinergic M_1_ to M_5_ receptors, dopamine D_1_ and D_2_ receptors
desloratadine	3.25	2.95	24.9	0.946	glycine receptor, cholinergic M_1_ to M_5_ receptors, serotonin 5-HT_2A/B_ receptors and transporter (SERT)
promethazine	4.54	3.63	6.5	0.867	Na^+^ and K^+^ channels, cholinergic M_1_ to M_5_ receptors, dopamine D_2_ receptor, histamine H2 receptor, adrenergic α-receptor
rupatadine	4.59	3.77	29.0	0.445	platelet activating factor receptor

a Predicted probability of a given compound being promiscuous, calculated by ADMETLab 3.0 software. Values closer to 1.00 mean high promiscuity.

b Retrieved from Guide to Pharmacology database.^28^

The main clinical outcome of the infection by *A. cantonensis* in humans is eosinophilic meningitis caused by the invasion of the larvae in the CNS.^8^ Therefore, agents that can cross the BBB are needed to adequately treat this condition. Since only cinnarizine and promethazine freely permeate the BBB,^31^ these two drugs are considered the most interesting agents for treating meningitis caused by *A. cantonensis* (Table 3). However, the promiscuity (lack of selectivity) associated with the tricyclic phenothiazine motif from promethazine is related to a significant blockade on ion channels, dopaminergic and adrenergic receptors, leading to effects such as arrhythmias, extrapyramidal motor effects, and orthostatic hypotension. Regarding this, cinnarizine can be considered the clinically most promising agent for further studies since its promiscuity is lower than for promethazine.

Overall, the findings suggest a novel avenue for anthelmintic development, whereby antihistamines exert their effects through mechanisms distinct from conventional anthelmintics. Further elucidation of the underlying pharmacological targets and mode of action of antihistamines against *A. cantonensis* may pave the way for the development of more effective therapeutic interventions against this parasite.

Finally, developing effective drugs against *A. cantonensis* faces significant challenges. The parasite’s complex life cycle, involving mollusk and vertebrate hosts, complicates identifying vulnerable stages for drug targeting. Additionally, the larvae’s ability to infiltrate the central nervous system makes drug delivery and efficacy difficult. The lack of comprehensive epidemiological data and underreporting of cases further hinder targeted treatment development. Current treatments are mainly supportive, and no anthelmintic drugs have proven effective, highlighting the urgent need for novel therapies and innovative approaches to combat this infection.

## 4 Conclusions

Infections caused by helminth parasites continue to impose significant morbidity on billions of individuals worldwide, yet the therapeutic options remain limited. Particularly concerning is the lack of effective and specific treatments for *A. cantonensis* infections despite their relatively low prevalence in humans. Our study addressed this critical gap by evaluating the susceptibility of *A. cantonensis* L1 larvae to clinically available antihistamines. This repurposing strategy holds immense potential for expediting the discovery of novel agents against *A. cantonensis*, serving as both inspiration and a practical pathway for the development of new drugs targeting this parasite.

In summary, this study represents an initial exploration into the anthelmintic potential of clinically available antihistamines against *A. cantonensis*. To our knowledge, this is the first report documenting the effects of these drugs on this parasite, marking a significant contribution to the field. Building upon these findings, efforts are underway to synthesize novel compounds based on these antihistamines as prototypes. These endeavors aim to elucidate the structure–activity relationships and optimize the anthelmintic efficacy of these compounds, thereby advancing the quest for effective treatments against *A. cantonensis* infections.

## 3 Materials and Methods

### 3.1 Drugs and Reagents

Commercially sourced antihistamine drugs, as detailed in Table 1, were acquired in pharmaceutical-grade purity from Sigma-Aldrich (St. Louis, MO), Cayman Chemical (Ann Arbor, MI), and Toronto Research Chemicals (Toronto, Ontario, Canada). RPMI 1640 culture medium and penicillin G/streptomycin solutions (10,000 units/mL penicillin G sodium salt, 10 mg/mL streptomycin sulfate) were procured from Vitrocell (Campinas, SP, Brazil), while dimethyl sulfoxide (DMSO) was acquired from Sigma-Aldrich. On the day of experimentation, drugs were freshly prepared by accurately weighing and dissolving them in DMSO, with consideration given to the weight and molecular weight of each compound to achieve a stock concentration of 10 mM.

### 3.2 Animals and Parasites

The life cycle of *A. cantonensis* (NPDN-AC strain) was maintained through a passage in *Biomphalaria glabrata* snails and Wistar rats (*Rattus norvegicus*) at the Research Center on Neglected Diseases (Guarulhos University, SP, Brazil). Both snails and rodents were housed under controlled environmental conditions (25 °C; 50% humidity), with ad libitum access to water and standard rodent chow.

### 3.3 Larvae Isolation

*A. cantonensis* first-stage larvae (L1) were isolated from rat feces following Rugai’s traditional method.^37^ Briefly, fecal samples collected 2 months postinfection were suspended in RPMI 1640 medium supplemented with 1% (v/v) penicillin/streptomycin solution and centrifuged at 300*g* for 4 min. Following the third wash in culture medium containing antibiotics, larvae were quantified and transferred to culture plates for anthelmintic assays.

### 3.4 Antiparasitic Assay

*In vitro* drug testing was conducted following established protocols for anthelmintic assays.^14^*A. cantonensis* L1 were transferred to 96-well culture microplates at a density of 100 larvae per well and maintained in RPMI 1640 medium supplemented with 1% (v/v) penicillin/streptomycin solution. Initial drug concentrations of 50 μM were utilized, and compounds demonstrating an effect exceeding 60% after 24 h postexposure underwent serial dilution in medium (50 to 1.56 μM). The final DMSO concentration in plates was maintained at 0.1% v/v, with wells containing the highest DMSO concentration in the medium serving as controls. Each drug concentration was tested in triplicate, with experiments repeated thrice. Larval viability was assessed immediately post drug addition (time 0) and after 2, 6, 12, and 24 h using an inverted microscope (BEL Engineering INV100, Monza, MB, Italy) equipped with a BEL Engineering ultrahigh definition (UHD) camera and a 48 in. 4K-UHD monitor system (LG Electronics, Taubaté, SP, Brazil).^38^ Larval motility, scored for an effect ≥60%, was categorized as follows: 1 (immotile), 2 (intermittent head or tail shaking), 3 (sluggish and motile), and 4 (highly active and motile).^14^ Images were captured using a Motic AE2000 inverted microscope (Vancouver, Canada) equipped with a Motic ultrahigh definition (UHD) camera.

### 3.5 Data Analysis

Each assay comprised triplicate tests, with 100 larvae per replicate, totaling 300 larvae for each concentration tested or control, and repeated at least thrice on different days. Mean responses and standard errors of the mean were calculated for each drug concentration by averaging across worms. Larval motility scores were determined by counting the number of larvae for each drug concentration and calculating the percentage of larvae exhibiting motility.^14^ For confirmation of EC_50_ values, a differential staining technique using propidium iodide penetration as the indicator of death was employed.^39^ The larvae were analyzed using a fluorescence inverted biological microscope INV100-FL (BEL Engineering). Concentration–response curves were generated using nonlinear regression in GraphPad Prism software 8.0, with EC_50_ values and respective 95% confidence intervals estimated as described previously.^40,41^

### 3.6 Ethics

All experimental procedures adhered to protocols approved by the Committee for the Ethical Use of Animals in Experimentation at Guarulhos University (Guarulhos, SP, Brazil; protocol ID 064/24).

### 3.7 Physicochemical and Molecular Descriptor Analysis

The active drugs cinnarizine, desloratadine, promethazine, and rupatadine were subjected to *in silico* calculation of their physicochemical, molecular, and pharmacokinetic properties in the ADMETlab 3.0 software (https://admetlab3.scbdd.com).^42^ The software uses information from topological and prediction models to estimate several properties. The selected descriptors in the present work were calculated *n*-octanol/water partition coefficient (*c* log *P*) and in physiological pH (*c* log *D*_7.4_), topological polar surface area (TPSA) and the promiscuity index. The data is presented in Table 3.
